# Supplementary material for: Arp2/3 and type-I myosins control chromosome mobility and end-resection at double-strand breaks in S. cerevisiae
Source: Nat Commun. 2025 Aug 5;16:7212. doi: 10.1038/s41467-025-62377-7 (PMC12325611; doi:10.1038/s41467-025-62377-7)
Supplement: Supplementary file 1 — Supplementary Information [file 41467_2025_62377_MOESM1_ESM.pdf]

Supplemental Figures

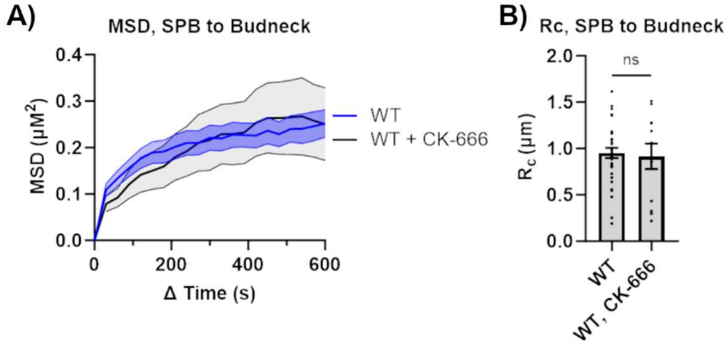

Supplementary Figure 1. Mobility of spindle pole bodies relative to the bud neck in response to CK-666

A. MSD analysis (Δt = 30s) of both SPBs relative to the bud neck in FZ015 (WT) (n=36) 3 h after Gal-HO induction. CK-666 (100 µm) (n=12) was added 20 min before imaging. Imaging and analysis done as described in (1C). Mean ± SEM is shown. Source data are provided as a Source Data file.

B. Bar graph of the Rc from MSD analysis of strains in (A). Statistical analysis for the radius of confinement derived in PRISM using a one-way Anova (ns $p \geq 0.05$, *$p < 0.05$, **$p < 0.01$, and ***$p < 0.001$). See **Supplementary Data 1** and **2** for significance and R_C values. Mean ± SEM is shown. Source data are provided as a Source Data file.

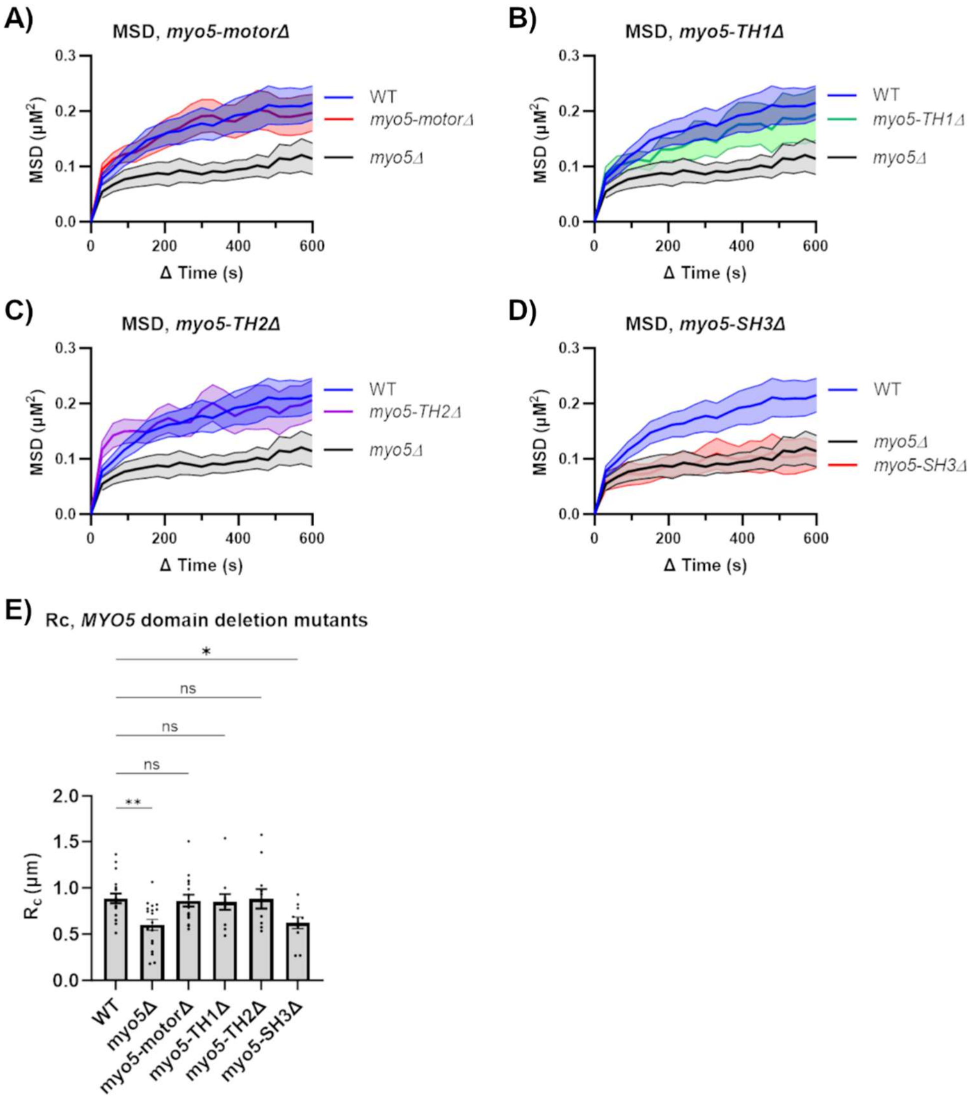

Supplementary Figure 2. MSD of a DSB in *MYO5* domain deletion mutants

A. MSD analysis (Δt=30s) of a DSB in FZ015 (WT) (n=20), FZ034 (*myo5*Δ) (n=18), and FZ048 (*myo5-motor*Δ) (n=16) 3 h after Gal-HO induction. Imaging and analysis done as described in (1C). Mean ± SEM is shown. Source data are provided as a Source Data file.
B. MSD analysis (Δt=30s) of a DSB in FZ054 (*myo5-TH1*Δ) (n=11). Imaging and analysis done as described in (1C). Mean ± SEM is shown. Source data are provided as a Source Data file.
C. MSD analysis (Δt=30s) of a DSB in FZ055 (*myo5-TH2*Δ) (n=11). Imaging and analysis done as described in (1C). Mean ± SEM is shown. Source data are provided as a Source Data file.
D. MSD analysis (Δt=30s) of a DSB in FZ056 (*myo5-SH3*Δ) (n=11). Imaging and analysis done as described in (1C). Mean ± SEM is shown. Source data are provided as a Source Data file.
E. Bar graph of the Rc from MSD analysis of strains in (A-D). Statistical analysis for the radius of confinement derived in PRISM using a one-way Anova (ns $p \geq 0.05$, *$p < 0.05$, **$p < 0.01$, and ***$p < 0.001$). See **Supplementary Data 1** and **2** for significance and R_C values. Mean ± SEM is shown. Source data are provided as a Source Data file.

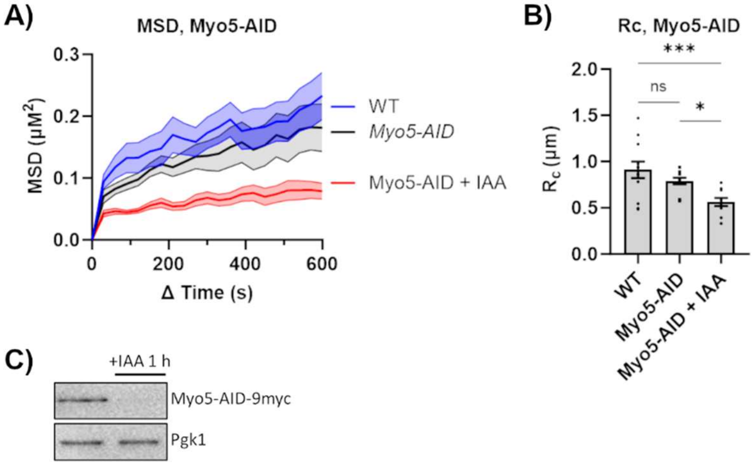

Supplementary Figure 3. MSD of a DSB in a Myo5-AID mutant

A. MSD analysis (Δt = 30s) of DSB relative to the SPB in FZ015 (n=12), FZ065 (Myo5-AID) (n=11), and FZ065 with IAA (Myo5-AID + IAA) (n=10) 3 h after Gal-HO induction. IAA (1 mM) was added 1 h before image collection. Imaging and analysis done as described in (1C). Mean ± SEM is shown. Source data are provided as a Source Data file.
B. Bar graph of the R_C from MSD analysis of strains in (A). Statistical analysis for the radius of confinement derived in PRISM using a one-way Anova (ns $p \geq 0.05$, *$p < 0.05$, **$p < 0.01$, and ***$p < 0.001$). See **Supplementary Data 1** and **2** for significance and R_C values. Mean ± SD is shown. Source data are provided as a Source Data file.
C. Western blot analysis of Myo5-AID ± auxin (IAA) (1 mM). IAA or an equivalent amount of 200 proof ethanol was added 2 h after adding galactose. Mouse α-Myc probed for Myo5-AID. Rabbit α-Rad53 probed for Rad53 phosphorylation. Mouse α-Pgk1 was probed as a loading control. Source data are provided as a Source Data file.

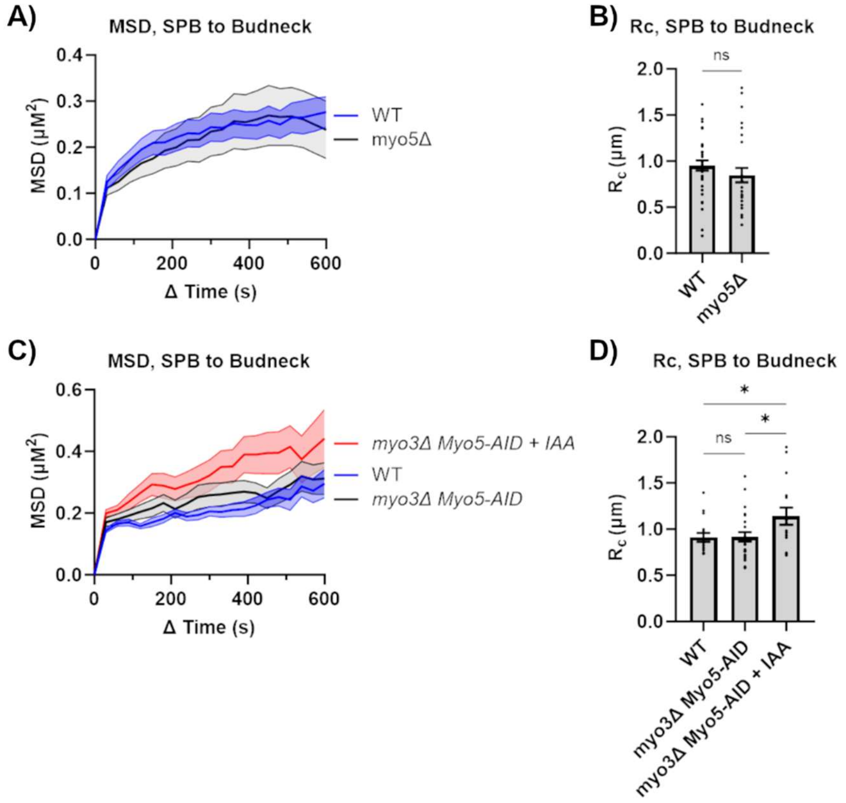

Supplementary Figure 4. MSD of the SPB in a *myo3Δ* Myo5-AID strain ± IAA

A. MSD analysis ($\Delta t = 30s$) of both SPBs relative to the bud neck in FZ015 (WT) (n=36) and FZ034 (*myo5Δ*) (n=28). Imaging and analysis done as described in (1C). Mean ± SEM is shown. Source data are provided as a Source Data file.
B. Bar graph of the Rc from MSD analysis of strains in (A). Statistical analysis for the radius of confinement derived in PRISM using a t test (ns $p \geq 0.05$, *$p < 0.05$, **$p < 0.01$, and ***$p < 0.001$). See **Supplementary Data 1** and **2** for significance and R_C values. Mean ± SEM is shown. Source data are provided as a Source Data file.
C. MSD analysis ($\Delta t = 30s$) of both SPBs relative to the bud neck in FZ015 (WT) (n=16), FZ074 (*myo3Δ* Myo5-AID) (n=26), and FZ074 with IAA (*myo3Δ* Myo5-AID + IAA) (n=16) 3 h after Gal-HO induction. Imaging and analysis done as described in (1C). Mean ± SEM is shown. Source data are provided as a Source Data file.
D. Bar graph of the Rc from MSD analysis of strains in (C). Statistical analysis for the radius of confinement derived in PRISM using a one-way Anova (ns $p \geq 0.05$, *$p < 0.05$, **$p < 0.01$, and ***$p < 0.001$). See **Supplementary Data 1** and **2** for significance and R_C values. Mean ± SEM is shown. Source data are provided as a Source Data file.

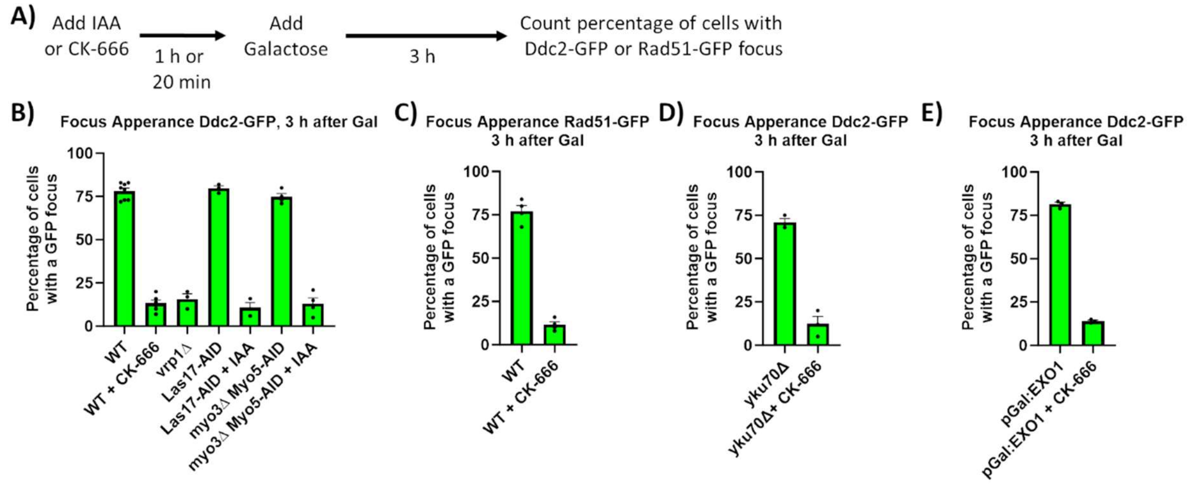

Supplementary Figure 5. Percentage of cells with a Ddc2-GFP or Rad51-GFP focus 3 h after DSB induction

A. Diagram of the experimental setup for measuring Ddc2-GFP or Rad51-GFP foci 3 h after DSB induction. Arp2/3 activity was inhibited either by CK-666 (100 µM) treatment 20 minutes before DSB induction or by adding IAA (1 mM) to degrade AID-tagged proteins 1 h before DSB induction.
B. Percentage of cells with a Ddc2-GFP focus 3 h after DSB induction in FZ015 (WT), FZ071 (*vrp1*Δ) FZ075 (Las17-AID), and FZ074 (*myo3*Δ Myo5-AID). CK-666 (100 µM) was added 20 min before galactose to WT and IAA (1 mM) was added to Las17-AID and *myo3*Δ Myo5-AID 1 h before adding galactose. Cells were collected 3 h after adding galactose. Cells were imaged with a 488 nm laser taking optical stacks of 1.5 µm with step size of 300 nm. Mean ± SEM is shown. Source data are provided as a Source Data file.
C. Percentage of cells with a Rad51-GFP focus 3 h after DSB induction in FZ012. CK-666 (10µM) was added 20 min before galactose. Sample collection and imaging was done as described in (B). Mean ± SEM is shown. Source data are provided as a Source Data file.
D. Percentage of cells with a Ddc2-GFP focus 3 h after DSB induction in FZ028 (*yku70*Δ) ± CK-666. CK-666 was added 20 min before galactose. Sample collection and imaging was done as described in (B). Mean ± SEM is shown. Source data are provided as a Source Data file.
E. Percentage of cells with a Ddc2-GFP focus 3 h after DSB induction in FZ201 (pGal:*EXO1*). CK-666 (10 µM) was added 20 min before galactose to WT. Cells were collected 3 h after adding galactose. and imaging was done as described in (B). Mean ± SEM is shown. Source data are provided as a Source Data file.

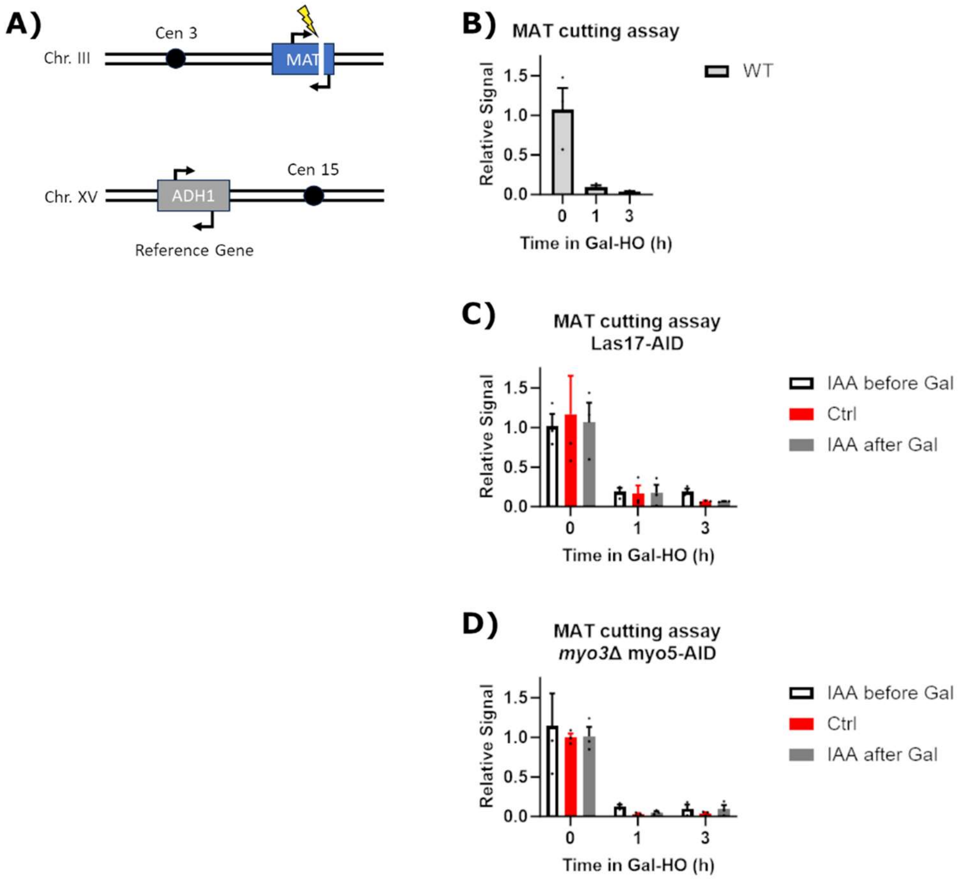

Supplementary Figure 6. Blocking Arp2/3 activity does not affect cutting by Gal-HO

A. Model of *MAT* cutting assay to determine Gal-HO cutting of the *MAT* locus on chromosome III. Primers flank the HO-cut site in the *MAT* locus. A second set of primers in *ADH1* on chromosome XV were used as a control.
B. Cutting assay of FZ041 (WT) shows that within 1 h after adding galactose. Mean ± SD shown. Source data are provided as a Source Data file.
C. Cutting assay of FZ075 (Las17-AID) shows that within 1 h after adding galactose. IAA was added either 1 h before or 1 h after adding galactose. Mean ± SD shown. Source data are provided as a Source Data file.
D. Cutting assay of FZ074 (*myo3Δ* Myo5-AID) shows that within 1 h after adding galactose. IAA was added either 1 h before or 1 h after adding galactose. Mean ± SD shown. Source data are provided as a Source Data file.

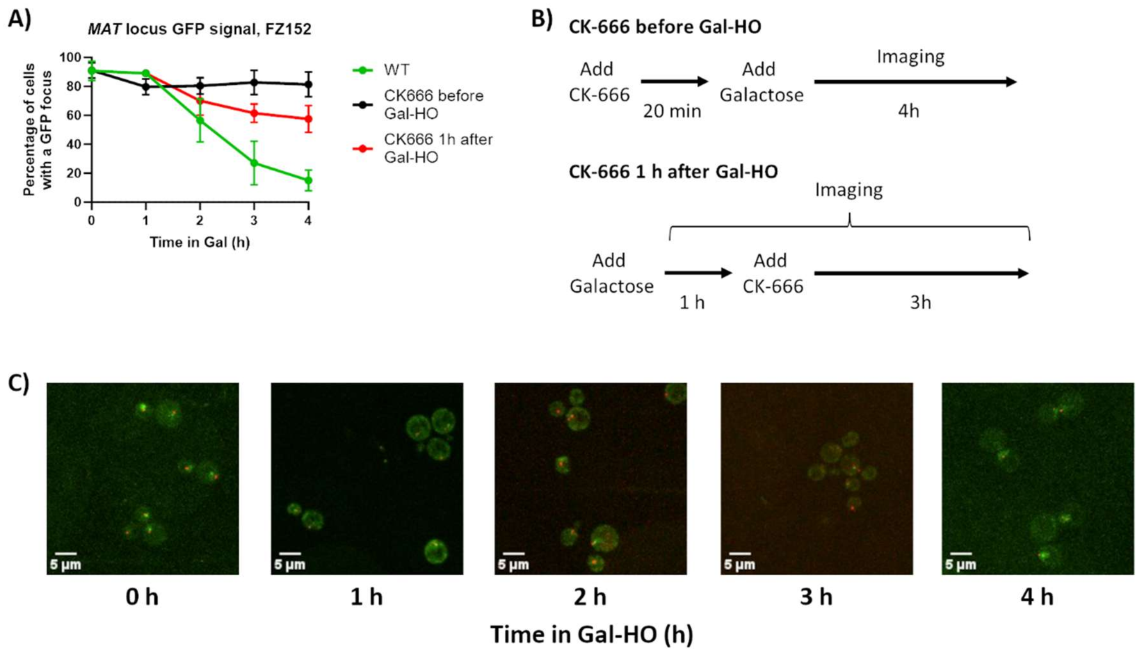

Supplementary Figure 7. Percentage of cells with a GFP-LacI focus after DSB induction

A. Percentage of cells with a GFP-LacI/*lacO* focus in FZ152. CK-666 (10 µM) was added either 1 h before galactose (black) or 2 h after adding galactose (red). Untreated cells are shown in green. Samples were collected every hour after adding galactose for up to 4 h. Cells were imaged with a 488 nm laser taking optical stacks of 1.5 µm with step size of 300 nm. Source data are provided as a Source Data file.
B. Experimental setup to measure the percentage of cells in the strain FZ152 with a GFP-LacI focus. CK-666 (100 µM) was added either 20 minutes before galactose or 1 h after galactose.
C. Example images from FZ152 0, 1, 2, 3, 4 h after DSB induction with Gal-HO. Mean ± SEM is shown. Source data are provided as a Source Data file.

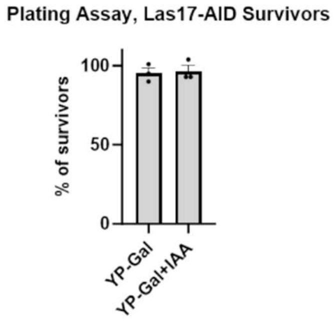

Supplementary Figure 8. Percentage of *MAT*a-inc Las17-AID survivors regrown on YP-Gal plates

Survival assay of FZ177 Las17-AID survivors from YP-Gal and YP-Gal+IAA plates regrown on YPD and YP-Gal plates. Graph shows the number of colonies on YP-Gal plates divided by the number of colonies on YPD plates. See **Methods** for more details. Mean ± SEM shown. Source data are provided as a Source Data file.

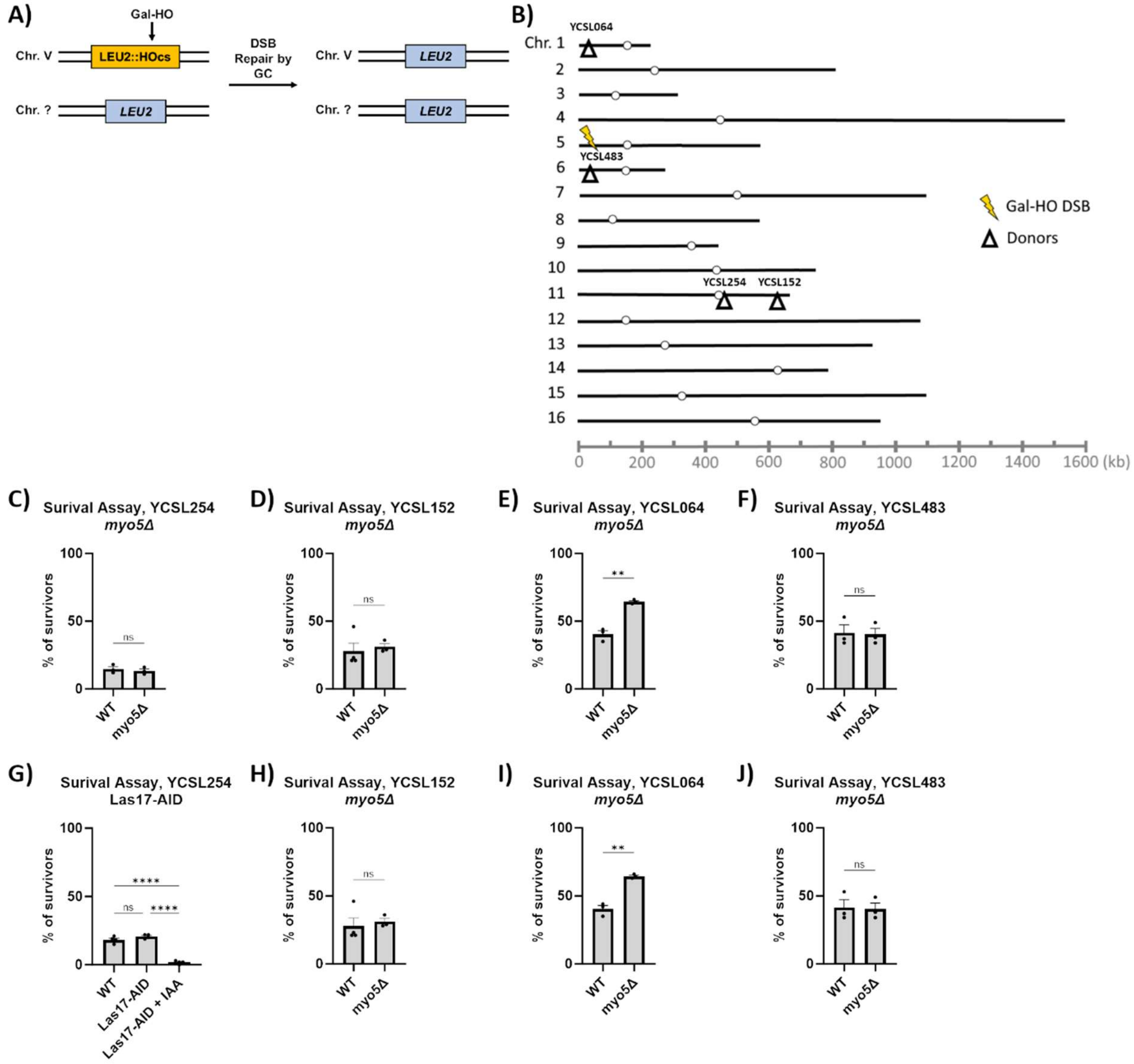

Supplementary Figure 9. The effect of donor position on repair by gene conversion

A. Model of repair strains with an HO-cut site in *LEU2* on chromosome 5 (*LEU2::HOcs*). Donors are located at different locations in the genome. DSB in *LEU2::HOcs* is repaired with an ectopic copy of *LEU2*.
B. Locations of the *LEU2* donors and the strain names. The *LEU2::HOcs* is located on chromosome 5.
C. Survival assay of YCSL254 wildtype and *myo5*Δ strains. ~200-100 cells were plated on YPD and YP-Gal plates in triplicate. The percentage of survivors was calculated by dividing the average survivors on YP-Gal plates divided by the average number of colonies on YPD plates. Statistical analysis for the percentage of survivors was conducted with an unpaired t-test. See **Supplementary Data 3** for significance. Mean ± SEM is shown. Source data are provided as a Source Data file.
D. Survival assay of YCSL154 wildtype and *myo5*Δ strains. Plating performed as in (C). Statistical analysis for the percentage of survivors was conducted with an unpaired t-test. See **Supplementary Data 3** for significance. Mean ± SEM is shown. Source data are provided as

a Source Data file.

E. Survival assay of YCSL064 wildtype and *myo5*Δ strains. Plating performed as in (C). Statistical analysis for the percentage of survivors was conducted with an unpaired t-test. See **Supplementary Data 3** for significance. Mean ± SEM is shown. Source data are provided as a Source Data file.

F. Survival assay of YCSL483 wildtype and *myo5*Δ strains. Plating performed as in (C). Statistical analysis for the percentage of survivors was conducted with an unpaired t-test. See **Supplementary Data 3** for significance. Mean ± SEM is shown. Source data are provided as a Source Data file.

G. Survival assay of YCSL254 wildtype and Las17-AID ± IAA. ~200-100 cells were plated on YPD, YP-Gal, and YP-Gal+IAA plates. The percentage of survivors was calculated by dividing the average number of survivors on YP-Gal and YP-Gal+IAA plates by the average number of colonies on YPD plates. Statistical analysis for the percentage of survivors was conducted with a one-way Anova. See **Supplementary Data 3** for significance. Mean ± SEM is shown. Source data are provided as a Source Data file.

H. Survival assay of YCSL154 wildtype and Las17-AID ± IAA. Plating performed as in (G). Statistical analysis for the percentage of survivors was conducted with a one-way Anova. See **Supplementary Data 3** for significance. Mean ± SEM is shown. Source data are provided as a Source Data file.

I. Survival assay of YCSL064 wildtype and Las17-AID ± IAA. Plating performed as in (G). Statistical analysis for the percentage of survivors was conducted with a one-way Anova. See **Supplementary Data 3** for significance. Mean ± SEM is shown. Source data are provided as a Source Data file.

J. Survival assay of YCSL483 wildtype and Las17-AID ± IAA. Plating performed as in (G). Statistical analysis for the percentage of survivors was conducted with a one-way Anova. See **Supplementary Data 3** for significance. Mean ± SEM is shown. Source data are provided as a Source Data file.
