# Supplementary material for: Arp2/3 and type-I myosins control chromosome mobility and end-resection at double-strand breaks in S. cerevisiae
Source: Nat Commun. 2025 Aug 5;16:7212. doi: 10.1038/s41467-025-62377-7 (PMC12325611; doi:10.1038/s41467-025-62377-7)
Supplement: Supplementary file 2 — Description of Additional Supplementary Files [file 41467_2025_62377_MOESM2_ESM.pdf]

Description of Additional Supplementary Files

File Name: Supplementary Data 1

Description: Statistical analysis of the difference in the radius of confinement to determine if there is a significant difference in the mobility of DSBs. One-way Anova or t-tests were used for this analysis. $p<0.05$ * $p<0.01$ ** $p<0.001$ *** Wildtype (WT) comparisons were made with data collected the same day. Dunnett test used for one-way Anova.

File Name: Supplementary Data 2

Description: Radius of confinement of DSBs derived from mean squared displacement analysis.

File Name: Supplementary Data 3

Description: Statistical analysis to determine if there is a significant difference in resection. Fresected (the percentage of resected DNA) was compared at 3 locations with different distances to the Gal-HO cut site in the *MAT* locus on chromosome III: 0.7 kb, 5 kb, and 10 kb. One-way Anova or t-tests were used for this analysis. $p<0.05$ * $p<0.01$ ** $p<0.001$ *** Dunnett test used for one-way Anova.

File Name: Supplementary Data 4

Description: Statistical analysis for the percentage of survivors in DSB repair assays. One-way Anova or t-tests were used for this analysis. $p<0.05$ * $p<0.01$ ** $p<0.001$ *** Dunnett test used for one-way Anova.

File Name: Supplementary Data 5

Description: List of strains.

File Name: Supplementary Data 6

Description: List of primers.

File Name: Supplementary Data 7

Description: List of plasmids.

File Name: Supplementary Data 8

Description: List of key resources.
