# Supplementary material for: Arp2/3 and type-I myosins control chromosome mobility and end-resection at double-strand breaks in S. cerevisiae
Source: Nat Commun. 2025 Aug 5;16:7212. doi: 10.1038/s41467-025-62377-7 (PMC12325611; doi:10.1038/s41467-025-62377-7)
Supplement: Supplementary file 11 — Reporting Summary [file 41467_2025_62377_MOESM11_ESM.pdf]

nature portfolio

Corresponding author(s): Jim Haber and Felix Zhou

Last updated by author(s): Jun 25, 2025

Reporting Summary

Nature Portfolio wishes to improve the reproducibility of the work that we publish. This form provides structure for consistency and transparency in reporting. For further information on Nature Portfolio policies, see our Editorial Policies and the Editorial Policy Checklist.

Statistics

For all statistical analyses, confirm that the following items are present in the figure legend, table legend, main text, or Methods section.

n/a	Confirmed	
☐	☒	The exact sample size (*n*) for each experimental group/condition, given as a discrete number and unit of measurement
☐	☒	A statement on whether measurements were taken from distinct samples or whether the same sample was measured repeatedly
☐	☒	The statistical test(s) used AND whether they are one- or two-sided<br>*Only common tests should be described solely by name; describe more complex techniques in the Methods section.*
☐	☒	A description of all covariates tested
☐	☒	A description of any assumptions or corrections, such as tests of normality and adjustment for multiple comparisons
☐	☒	A full description of the statistical parameters including central tendency (e.g. means) or other basic estimates (e.g. regression coefficient) AND variation (e.g. standard deviation) or associated estimates of uncertainty (e.g. confidence intervals)
☐	☒	For null hypothesis testing, the test statistic (e.g. *F*, *t*, *r*) with confidence intervals, effect sizes, degrees of freedom and *P* value noted<br>*Give P values as exact values whenever suitable.*
☒	☐	For Bayesian analysis, information on the choice of priors and Markov chain Monte Carlo settings
☒	☐	For hierarchical and complex designs, identification of the appropriate level for tests and full reporting of outcomes
☒	☐	Estimates of effect sizes (e.g. Cohen's *d*, Pearson's *r*), indicating how they were calculated

Our web collection on statistics for biologists contains articles on many of the points above.

Software and code

Policy information about availability of computer code

Data collection	Microscopy was done with a Nikon Elements AR software on a Nikon Ni-E upright microscope equipped with a 100× (NA, 1.45) oil immersion objectives, a Yokogawa CSU-W1 spinning-disk head, and an Andor iXon 897U EMCCD camera. Fluorophores were excited at 488nm (GFP) and 561nm (mCherry). qPCR experiments were conducted on a Biorad CFX384 Real-Time System C1000 Touch Thermal Cycler qPCR machine using Bio-Rad CFX Maestro 1.1 Version 4.1.2433.1219.
Data analysis	MATLAB 2023b with a custom program for mean squared displacement analysis was used to calculate the mean squared displacement curve and radius of confinement of double-stranded breaks. Code is provided on GitHub at DOI: 10.5281/zenodo.15742268. Information can be found in the Code Availability section of the paper.

For manuscripts utilizing custom algorithms or software that are central to the research but not yet described in published literature, software must be made available to editors and reviewers. We strongly encourage code deposition in a community repository (e.g. GitHub). See the Nature Portfolio guidelines for submitting code & software for further information.

Data

Policy information about availability of data

All manuscripts must include a data availability statement. This statement should provide the following information, where applicable:

- Accession codes, unique identifiers, or web links for publicly available datasets
- A description of any restrictions on data availability
- For clinical datasets or third party data, please ensure that the statement adheres to our policy

Data used to create the figures and tables in this paper have been provided in the Source Data file.

Research involving human participants, their data, or biological material

Policy information about studies with human participants or human data. See also policy information about sex, gender (identity/presentation), and sexual orientation and race, ethnicity and racism.

Reporting on sex and gender	N/A
Reporting on race, ethnicity, or other socially relevant groupings	N/A
Population characteristics	N/A
Recruitment	N/A
Ethics oversight	N/A

Note that full information on the approval of the study protocol must also be provided in the manuscript.

Field-specific reporting

Please select the one below that is the best fit for your research. If you are not sure, read the appropriate sections before making your selection.

☒ Life sciences ☐ Behavioural & social sciences ☐ Ecological, evolutionary & environmental sciences

For a reference copy of the document with all sections, see nature.com/documents/nr-reporting-summary-flat.pdf

Life sciences study design

All studies must disclose on these points even when the disclosure is negative.

Sample size	An n of 3 was used for each experiment with at least 3 biological replicates. For measuring mobility at least 10 cells were analyzed per condition.
Data exclusions	No data was excluded.
Replication	Videos for measuring double-strand break mobility were captured across at least 2 days to minimize variability in the day to day variances in microscopy conditions. For resection analysis, morphology analysis, and plating assays data was collected from 3 biological replicates in triplicate.
Randomization	Wildtype samples were collected each time experiments were conducted. For statistical analysis, only wildtype data collected from the same day as the different experimental conditions was used for comparison.
Blinding	Blinding was not relevant to this study.

Reporting for specific materials, systems and methods

We require information from authors about some types of materials, experimental systems and methods used in many studies. Here, indicate whether each material, system or method listed is relevant to your study. If you are not sure if a list item applies to your research, read the appropriate section before selecting a response.

Materials & experimental systems

n/a	Involved in the study
☐	☒ Antibodies
☒	☐ Eukaryotic cell lines
☒	☐ Palaeontology and archaeology
☒	☐ Animals and other organisms
☒	☐ Clinical data
☒	☐ Dual use research of concern
☒	☐ Plants

Methods

n/a	Involved in the study
☒	☐ ChIP-seq
☒	☐ Flow cytometry
☒	☐ MRI-based neuroimaging

Antibodies

Antibodies used

Mouse monoclonal anti-Rad53 AbCam Cat # ab166859 1:1000 dilution.
RRID:AB_2801547, Mouse monoclonal anti-myc AbCam Cat# ab16918; RRID:AB_30256 1:1000 dilution
Mouse monoclonal anti-Pgk1 AbCam Cat# ab32, RRID:AB_30359.1:1000 dilution

Validation

Antibodies were bought from AbCam and antibody validation can be found on product pages for each antibody.

Plants

Seed stocks

N/A

Novel plant genotypes

N/A

Authentication

N/A
